# Do Handling and Transport Stress Influence Adrenocortical Response in the Tortoises (*Testudo hermanni*)?

**DOI:** 10.1155/2014/798273

**Published:** 2014-02-20

**Authors:** Esterina Fazio, Pietro Medica, Giuseppe Bruschetta, Adriana Ferlazzo

**Affiliations:** Department of Veterinary Sciences, University of Messina, 98168 Messina, Italy

## Abstract

The goal of this study was to analyze circulating cortisol levels from tortoises (*Testudo hermanni*) to establish reference intervals and to develop guidelines for the interpretation of the effect of handling and transport stress. Blood samples were obtained from the caudal venous from 23 healthy juvenile tortoises (9 males and 14 females), aged 8–20 years, in basal condition, four weeks prior to and four weeks following handling and short transportation. The study was carried out on the experimental group: 10 tortoises, 4 males and 6 females, and on a control group: 13 tortoises, 5 males and 8 females. Compared to basal values, circulating cortisol concentrations was higher after handling and transport (+286%; *P* < 0.001), with an increase of +246% (*P* < 0.001) in males, +236% (*P* < 0.005) in females, +370% (*P* < 0.005) in subjects aged 8–12 years, and +240% (*P* < 0.001) in subjects aged 13–20 years. These observations support the hypotheses that cortisol may act to mediate the effects of handling and transport stress in this species and that four weeks following handling and transport were insufficient to restore their homeostasis.

## 1. Introduction

Many endocrine responses in tortoises are very similar to those of other reptiles, even if they also show some specific aspects [1]. Several reptiles, including sea turtles and tortoises, are known to modify their stress response according to their body condition and analysis of blood constituents is useful in the diagnosis of health and disease status [2, 3] and has helped to identify stressed or ill tortoises [4–6].

Previous studies have described the effects of external and internal factors on the capacity of individuals to induce different physiological coping strategies in response to stressors. Changes of animal homeostasis induce a hormonal response that results in an increase of glucocorticoids, catecholamines, and other blood factors [7–9], necessary to restore their homeostasis.

Factors, such as disease and body condition, have been associated with significant changes in plasma corticosterone [10–13]. Measurement of plasma cortisol values is commonly used as a diagnostic technique to assess welfare or stress conditions of individual animals [14, 15]. However, there appears to be fragmentary information on reference intervals and physiological alterations in hormonal values in tortoises [16, 17] and in free-ranging desert tortoises [18]. In the last years the clinic reports, as repair and dressing of fractures, surgeries, shell diseases, and mycoplasmosis, related to cases suffered by Chelonia, were scarce and inadequate [19, 20]. Moreover, recently terrestrial tortoises and aquatic turtles are considered “company animals” and are often kept as pets; their numbers are increased, and the requests of routine veterinary checks are arisen [21, 22]. In addition, there are many occasions when it is necessary to transport the tortoises. This could be to transfer to a new home, even holiday boarding or a visit for routine veterinary checks. Tortoises, as all animals, can suffer from stress induced by a new environment much more quickly if their journey is planned with a little thought from their owner.

On this basis, the objective of the present study was to evaluate the effects of handling and transport stress on reference values of circulating cortisol concentrations in tortoises. Comparison between basal and posthandling and transport values could offer the opportunity to further our knowledge and improve the understanding of the adrenocortical response to stress and the healthy status of tortoises.

## 2. Materials and Methods 

### 2.1. Experimental Animals

The study was carried out on a total of twenty-three not hibernated tortoises (*Testudo hermanni*), in good health (9 males and 14 females), aged 8–20 years (10 aged 8–12 y and 13 aged 13–20 y), weighting 709 ± 144.97 g, the straight carapace length ranging from 13.50 to 18.50 cm (15.50 ± 1.41). On the basis of straight carapace length (SCL) the subjects were classified as juvenile tortoise (<40 cm). We examined circulating cortisol concentrations four weeks prior to and four weeks following handling and transport in experimental group (10 tortoises, 4 males and 6 females), and only following to handling in control group (13 tortoises, 5 males and 8 females). The body mass of the tortoises was measured to the nearest 10 g with 2 kg spring balance. Straight carapace length (cm) was measured with calipers for each tortoise. Gender was determined on the basis of presence of secondary external sex character.

The subjects were kept at the private home with natural light; they were fed with green vegetables, cooked potatoes and had free access to clean drinking water *ad libitum*.

A plastic and opaque container just slightly bigger than the tortoises was used and secured in the boot of car, ensuring it into van so that it cannot move around or shoot across the other side of the boot.

Each animal was given a thorough physical examination (examination of the oral and anal orifice, heart rate, respiratory rate, hydration state, body weight, and straight carapace length), and individuals did not shown signs of clinical illness (e.g., weight loss, hyperthermia, anorexia, and macroparasites). All tortoises after clinic check were defined in good health. Handling included weighing, measuring, and routine veterinary check.

### 2.2. Blood Sampling

Blood samples were collected by sterile syringe from the caudal venous sinus for each turtle with a 0.6 gauge (23G) needle, in basal condition (four weeks prior to handling and transport) and four weeks following to handling and short road transport of 40 km (duration of 90 minutes). The long interval between basal and posthandling and transport sampling (four weeks) was imposed by the tortoises' size and body weight. At their arrival at Veterinary Clinic (Messina, Italy) the subjects were submitted to clinic examination for evaluation of the general health status and then the subjects were submitted to blood sampling.

Blood samples were collected in both groups in spring (May/June) at 10.30 a.m. into lithium heparin tube (Venoject, Terumo, Belgium) and plasma was harvested and stored in Eppendorf tubes at −20°C until assayed for cortisol values.

### 2.3. Parameters and Methods

Plasma total cortisol concentrations were analysed in duplicate through a competitive enzyme assay (EIA, RADIM, Pomezia, Roma, Italy). During the first incubation, sample cortisol competed with the cortisol conjugated to horseradish peroxidase (HRPO) for binding to the specific sites of the antiserum coated on the wells. Following incubation, all unbound material was removed by aspiration and washing. The enzyme activity, which was bound to the solid phase, was inversely proportional to the cortisol concentration in calibrators and samples, which was evidenced by incubating the wells with a chromogen solution (tetramethylbenzidine, TMB) in a substrate buffer. Colourimetric reading was carried out using a spectrophotometer at 450 and 405 nm (Sirio S, RADIM/SEAC Co., Rome, Florence, Italy). The assay sensitivity was 5 ng/mL. The intra- and interassay coefficients of variation (CVs) were 4.6% and 6.9%, respectively.

### 2.4. Statistics

Data are presented as means ± standard deviation (SD). Significant differences between basal and posthandling and transport values were established using the Student's *t*-test for paired data. Significant differences between different genders, age, and groups (experimentals and controls) were established using the Student's *t*-test for unpaired data. The percentage difference between basal and posttransport values was calculated (Δ%). The level of significance was set at *P* < 0.05. All calculations were performed using the PRISM package (GraphPad Software Inc., San Diego, CA).

## 3. Results 

**Figure 1:**
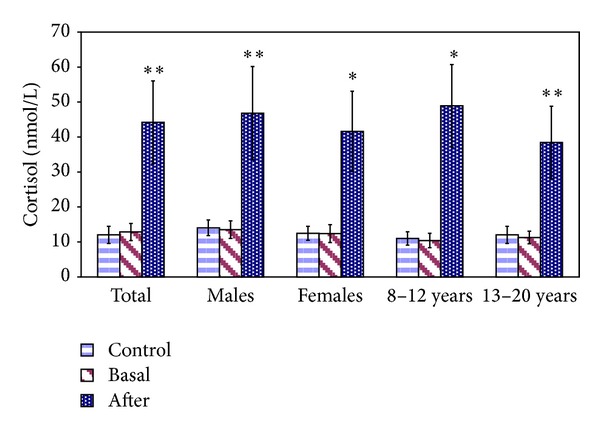
Circulating cortisol concentrations (M ± S.D.) of tortoises (*Testudo hermanni*) before and after to handling and transport stress versus basal: *P < 0.005; **P < 0.001.

Circulating cortisol determinations are reported in Figure 1. Basal cortisol concentrations, ranged between 9.79 and 16.52 nmol/L, and posthandling and transport concentrations, ranged between 31.12 and 59.84 nmol/L, were partially in agreement with physiological ranges previously reported in the literature in stressed and unstressed juvenile turtles [23].

Compared to basal condition, circulating cortisol concentrations was higher after transport (+244%; *P* < 0.001) in total subjects, with an increase of +246% (*P* < 0.001) in males, +236% (*P* < 0.005) in females, +370% (*P* < 0.005) in subjects aged 8–12 years, and +240% (*P* < 0.001) in those aged 13–20 years, respectively. No significant differences between cortisol basal concentrations of experimental group and control group were observed.

On the basis of different gender and age (Figure 1), no significant differences in basal cortisol concentrations between males and females and between tortoises aged 8–12 years and those aged 13–20 years were observed.

In addition, subjects aged 8–12 years showed no significant lower basal cortisol concentrations and no significant higher posttransport concentrations than tortoises aged 13–20 years.

## 4. Discussion

Comparison between our data and other results are somewhat limited due to potential differences between tortoise populations as well as to variations in analytical methods. Physiological endocrine values have not been established for most free-living tortoise populations. Researches are mainly focused on wild populations of the West African hinge-backed tortoise (*Kinixys erosa*), the free-ranging desert tortoise in the Mojave desert and in marginated tortoise (*Testudo marginata*) in different geographic environments [18, 22, 24] and in Mojave population of the desert tortoises (*Gopherus agassizii*) [25]. In addition, many researches reported corticosterone ranges because it is the major glucocorticoid in reptiles secreted from adrenocortical tissue [6, 8, 25–27] and the syndromes of glucocorticoid resistance were observed [28]. Cortisol and corticosterone are the main glucocorticoid hormones usually presented and discussed in the literature in terms of the mean responses to stress stimuli in most animals including reptiles, and they have been used as indicators to determine the duration and severity of stress [29–31]. Whilst the physiological functions of corticosterone have been more investigated in domestic and wild animals there are relatively insufficient descriptions of changes in serum cortisol concentrations in reptiles in general and tortoises in particular [23]. However, there are neither studies on changes in cortisol concentration relative to transport stress in tortoises nor data on how long this adrenocortical response could persist. Because handling tortoises to collect blood can induce stress that could influence their ability to detect a response to transport, we conducted this study to quantify the time required for *Testudo hermanni* to elicit elevated total cortisol concentrations after short road transport.

Hence, the comparisons of results obtained in this study with previously published data for tortoises revealed some discrepancies for circulating cortisol concentrations [23]. However, some differences may also possibly be explained by influence of individual variability (gender, age, and size), physiologic and breeding status [22, 24], or geographical location, habitat, seasonal variations [19, 32, 33], and immunological system [34].

The differences in cortisol values, as reported for corticosterone, could be indirectly due to intrinsic biological rhythms or could be directly determined by handling in tortoises [10, 12, 35, 36].

In addition, data obtained in healthy tortoises, without significant differences in basal condition between male and female cortisol values, confirm previous data described for corticosterone in *Gopherus polyphemus* [37]. In addition, previous studies [38–40] confirm higher basal cortisol concentrations in males than females. Data obtained confirm that males showed the earlier adrenocortical response to handling and transport stress compared to females [8]. Nevertheless, the significant differences between before and after cortisol concentrations reported in our study suggest that this hormone, namely the corticosterone, remains already significantly higher after 30 and 60 minutes after handling and release from restraint [36]. In addition, Cash et al. [26] noted that there was no change in glucocorticoids in wild red-eared slider turtles (*Trachemys scripta elegans*) in the first 10 minutes of capture, but concentrations significantly increased within 30 minutes. This lag in glucocorticoid response is consistent with previous research carry out on wild gopher tortoises (*Gopherus polyphemus*) [37, 40] and free-living loggerhead turtles (*Caretta caretta*) [10]. Plasma corticosterone did not increase significantly in ACTH-injected desert tortoises until 20 minutes postinjection, but there was significant increase 60 minutes postinjection, with an average increase of 392% (+/− 129%) [25].

Stress is generally assumed to be an inevitable outcome of transport because it requires the handling and movement of animals. Though, data obtained confirm that handling, confinement, and transport can affect metabolism and physical activity of tortoises [39, 41], resulting in the highest cortisol concentrations. Since the tortoises were submitted to blood sampling both four weeks prior to (in basal conditions) and four weeks following handling and transport, this aversive stimuli could be considered neutral. This hypothesis was confirmed by the absence of significant differences between control and basal cortisol values.

On the contrary, handling, confinement, and transport can be considered stressful, as confirmed by significant higher cortisol concentrations four weeks following experimental protocols than basal values.

On the basis of different age, subjects aged 8–12 years showed lower basal cortisol concentrations but higher posttransport concentrations than tortoises aged 13–20 years. These differences, even if not significant, can be interpreted in terms of different stress responses, according to different age, as reported by Gregory and Schmid [36]. Subjects aged 13–20 years seemed to be more protected against transport stress.

On the basis of different gender, the results obtained in females did not explain the effect of handling and transport on cortisol changes, since the cortisol concentration averages in tortoises were included in subjects ranged 8–12 years; in fact, the subjects aged 8–12 years included the fourteen females. An alternative explanation for the presence of the highest basal cortisol concentrations in males in the present study could be proper the age of these specimens, ranged between 13 and 20 years. The absence of significant differences of cortisol concentrations between males and females confirms previous data observed in free-living turtles for corticosterone secretion in response to capture and handling [26].

The results obtained do not exclude that the highest cortisol concentrations in basal conditions of males and subjects aged 13–20 years may have determined the lowest cortisol concentrations after handling and transport because the secretions of this hormone is yet high and near the physiological range reported for tortoises. On the other hand, the highest cortisol values observed in males and in subjects aged 8–12 years after transport could be explained on the basis of a major secretion or reserve of cortisol concentrations. It is possible that stress conditions were added by handling, confinement, and transport, which were always performed in tortoises only four weeks following to handling and transport.

In conclusion, the significant differences observed between, before, and after determinations in healthy tortoises, in accordance with different gender, suggest a similar response to handling and transport stress; however, the cortisol increase was more representative in males (+246%) than in females (+236%). On the contrary, the significant increase of cortisol concentrations in tortoises aged 8–12 years suggest that the younger subjects response with a significant secretion of this hormone because probably handling and transport may be mainly perceived as stressful. It appears that these results comprise a variety of stressors, as handling (including weighing and measuring, routine veterinary check), restraint, confinement, transport, while blood sampling may have elicited the stress response. Our data suggest that blood sampling per se is not an acutely stressful event in juvenile tortoises; hence, it possible that tortoises could exhibit a diminished responsiveness of central and peripheral catecholaminergic systems to acute stress, simultaneously with progressive hypothalamic corticotrophin-releasing hormone deficiency, as reported in nonsenescent older rats [42] and horses [43, 44].

## 5. Conclusion

In the present study we tested the hypothesis that tortoises submitted to handling and transport stress possess a hyperresponsive hypothalamo-pituitary-adrenal (HPA) axis in relation to turtles. In fact previous studies showed that the adrenocortical response of sea turtles appears to be substantially slower than that of other vertebrates [45–47].

The veterinary check could be a concomitant cause of additional stress because every subject was submitted to the same handling by the same operator. Because the rate of increase in circulating hormone concentrations during handling is considered a marker of the sensitivity of the HPA axis also in lizards and turtles and can be used to evaluate an animal's endocrine sensitivity to stress [8, 23, 26, 27], monitoring tortoise blood cortisol changes can be a way to evaluate the physiologic and health status of this populations (*Testudo hermanni*). In addition, this hormone may be a useful indicator of the environmental status, because these species are very sensitive to habitat changes [33].

The appropriate sample handling in order to deliver meaningful diagnostic data and the establishment of baseline blood cortisol profiles for healthy tortoises are a priority to provide accurate and reproducible quantitative analyses in this species.

Despite the lack of the number of subjects, this study extends our knowledge of the effects of handling and transport of tortoises and raises interesting questions about the adrenocortical response of these stressful stimuli in this species.

Previous work on corticosterone responses to capture and restraint in turtles and tortoises showed different blood sampling time, ranging between 30 minutes [26] and 24 hours [48]. In conclusion, although circulating cortisol or corticosterone concentrations are the golden standard to study the hypothalamic-pituitary-adrenal (HPA) axis, many complementary investigation tools have been developed to overcome the various problems related to blood sampling time.
